# Clear Aligners and Miniscrews in a Scissor Bite Adult Treatment

**DOI:** 10.1155/2024/8841829

**Published:** 2024-02-23

**Authors:** Teresa Pinho, Duarte Rocha, Sara Gonçalves, Maria Luís Martins

**Affiliations:** ^1^UNIPRO—Oral Pathology and Rehabilitation Research Unit, University Institute of Health Sciences (IUCS), CESPU, 4585-116 Gandra, Portugal; ^2^IBMC-Instituto Biologia Molecular e Celular, i3S-Inst. Inovação e Investigação em Saúde, Universidade do Porto, Porto, Portugal

## Abstract

Scissor bite does not correct spontaneously. It gradually worsens by overeruption, negatively affecting masticatory function. It is intended with this manuscript to evaluate the different treatment strategies to correct this malocclusion in adult patients, exploring treatment with clear aligners, bite ramps, and MS (miniscrews), especially in this case of a patient with unilateral right scissor bite, with high dental compensation in the three planes of space, asymmetrical sagittal dental position, overeruption on the scissor bite condition, and a high mandibular arch constriction and maxillary expansion. A comprehensive literature research was performed from 2002 until March 2023. PubMed and BVS databases were used, with the following keywords: “scissor bite OR brodie bite” AND “malocclusion” AND “treatment OR correction OR therapeutics”. Since correcting skeletal asymmetries after the growth completion is challenging, adult patient cases often involve a combined orthodontic-surgical approach. In the present clinical case, the severe limitations to decompensating tooth positions for a surgical treatment, with the necessity to perform lower asymmetric extraction and a must longer orthodontic treatment, were the major reasons to avoid the surgical approach, after the scissor bite correction. In spite of this, the efficiency of the clear aligners and auxiliaries like bite ramps, MS, and elastics in successfully correcting a complex scissor bite in an adult patient was demonstrated, with significant esthetic and functional commitment, demonstrated by the case reliability PAR (peer assessment rating) index.

## 1. Introduction

Scissor bite can be described as a malocclusion in which the mandible arch was contracted within the maxillary arch. This is a condition that can be caused by a narrow mandible and/or a large maxilla [1, 2]. This malocclusion can be related to functional problems such as asymmetries, occlusal interferences, mandibular rotation, and occlusal plane inclination, affecting jaw growth and mastication [1, 3, 4].

Keep the scissor bite untreated, negatively affects masticatory function and can lead to temporomandibular joint abnormalities [5]. Patients are also often characterized by having struggle in lateral functional movements [6].

As the jaw growth slows down as the patient ages, correcting a scissor bite becomes more difficult. Due to complications in correcting skeletal asymmetries after growth completion, most of the adult patients' cases involve a combined orthodontic-surgical treatment. However, surgical approaches are sometimes not easily accepted by patients [6–9].

On the other hand, less invasive coadjuvant procedures, such as MS (miniscrews) associated or not with bite planes, are particularly appropriate for severe scissor bite treatment, even in camouflage orthodontic treatments, in adult patients [6, 10].

The implementation of clear aligners in the past years has updated the orthodontics field since these appliances offer an inviting alternative to a society in which esthetics and comfort are values of great interest [10].

This article intends to explain the different approaches for the correction of scissor bite in an adult patient with a complex malocclusion treated with aligners and auxiliaries (bite ramps, MS and elastics). PAR (peer assessment rating) index was taken to evaluate the case reliability [11].

## 2. Case Report

### 2.1. Diagnosis and Etiology

**Figure 1:**
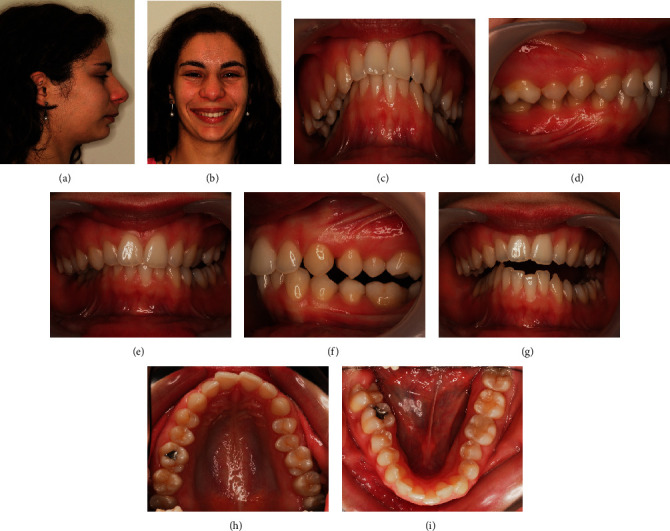
24-year-old female patient with a unilateral right scissor bite, with dental overeruption and very negative torque in the mandibular arch bilaterally: (a) profile photo; (b) smile; (c) overjet view; (d–f) intraoral photos in maximum intercuspation/centric relation; (g) protrusive guide; (h/i) occlusal photos.

A 24-year-old female patient presented with total unilateral right scissor bite, with dental overeruption and very negative torque in the mandibular arch, bilaterally (Figure 1).

This malocclusion etiology was primarily dental, with many compensations in the 3 planes of space and inherent skeletal repercussions such as marked compression of the mandibular arch and facial asymmetry with chin deviation. The patient has a convex profile (Figure 1(a)).

The dental asymmetry was in all 3 planes of space, being evident with the teeth overeruption on the entire side of the scissor bite. There was a significant asymmetrical sagittal positioning of the lower canines. Through intraoral examination, it was verified a right molar and canine Class II with an increased deep bite and a left Class III relationship with an open bite. A deep bite with pronounced *cant* was noted in the lower occlusal plane, on the side of the scissor bite (Figure 1).

**Figure 2:**
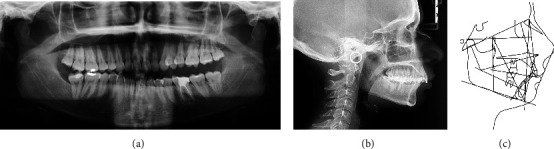
(a) Initial panoramic X-ray, (b) teleradiograph, and (c) cephalometry.

A panoramic radiograph revealed the presence of all wisdom teeth except on the lower right side. In a first stage, tooth 1.8 was maintained despite not having its opponent, to promote anchorage to move the adjacent teeth with more predictability (Figure 2). Later, the tooth was extracted.

In the cephalometric analysis, the following could be established: an hypodivergent biotype (Frankfort-mandibular plane angle, FMA = 20.7°; norm = 25 + 3); pronounced skeletal Class II (convexity of the Apoint = 9.5 mm; norm = 2 + 2); Class II at the ANB angle level (10.1°; norm = 3 + 2); pronounced alveolar Class II (distance A‐B = 13.3 mm; norm = 5 + 1), with promaxilla (SNA = 86.7°; norm = 82 + 2°) and retromandible (SNB angle = 76.5°; norm = 80 + 2°); and decreased interincisal angle (126.2°; norm = 132 + 6°), with accentuated retroclination of the upper incisors (UI/NA = 8.9°; norm = 22 + 2°) and proclination of the lower incisors (mandibular incisor to mandibular plane angle, IMPA = 109.4°; norm = 89.5 + 2.5°) (Figure 2).

### 2.2. Treatment Objectives

1. Scissor bite and deep bite correction, achieving a stable occlusal relationship with occlusal contacts and function
2. Constriction of maxillary arch especially on the right side and bilateral mandibular arch expansion, to correct the crowding and the high negative torque
3. The facial objectives were to improve the smile as much as possible, correcting the *cant* of the occlusal plan, considering that this was an orthodontic camouflage

### 2.3. Treatment Alternatives

Since the patient had a skeletal mandibular retrusion, associated with a convex profile, an orthodontic-surgical treatment was primarily suggested as the ideal option. Given the asymmetrical dental component in the lower arch with evident asymmetry in canine positioning, a premolar extraction or a high marked distalization on the 3rd quadrant (which is the side of the Class III relation) would be necessary. However, this procedure would complicate the lower arch expansion and so the scissor bite correction, which was required before the surgical approach with mandibular advancement. Therefore, the priority was to treat the transverse and overeruption problems associated with the scissor bite condition, even with the sagittal asymmetric tooth position.

### 2.4. Treatment Progress

**Figure 3:**
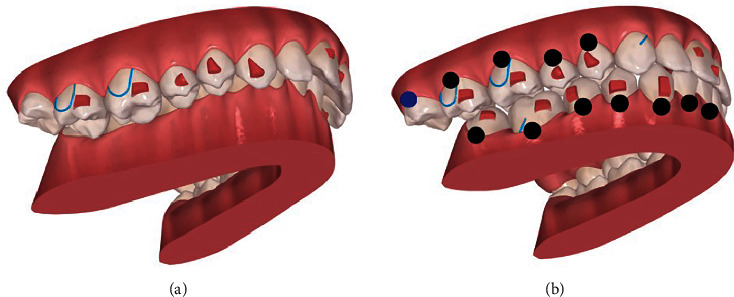
Initial ClinCheck® (version 6.0): (a) beginning; (b) planned. Blue dots—moderate movements; black dots—complex movements.

**Figure 4:**
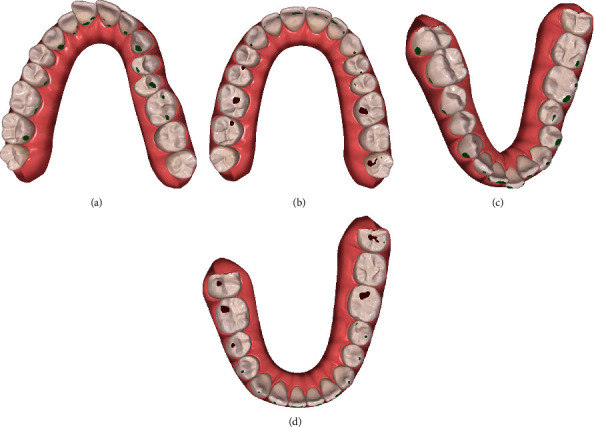
Initial ClinCheck®: (a/c) initial and (b/d) planned occlusal contacts.

**Figure 5:**
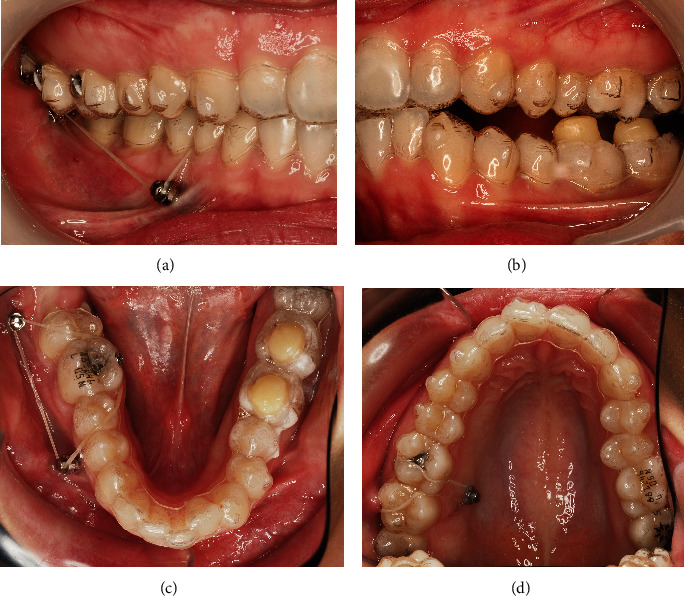
Start of treatment with placement of aligners that were cut into the occlusal surface of the 3rd quadrant, to attach bite ramps to allow the unblocking of the right side. Correction of the scissor bite with MS using elastics and buttons; MS in the mandible: retromolar trigone and mandibular inter-radicular between teeth 44 and 45; miniscrew in the maxilla: palatal inter-radicular between teeth 16 and 17.

The patient's treatment approach included the Invisalign® system (Align Technology, San Jose, California, USA) with inter-radicular MS between the upper right molars, connected to buccal buttons, for maxilla compression (Figures 3–5). On the other hand, for mandible expansion, two MS were placed: one in the retromolar trigone; and one inter-radicular between the right premolars, buccally, associated with elastics to lingual buttons on teeth 47, 46, 45, and 44 (Figure 5). In the 1st quadrant, at the beginning, the patient used the elastic under the aligner, since in a first stage there was some difficulty in the day-to-day life to remove it whenever she needs to remove the aligner to eat.

Elastics and MS are near the dental surface, not causing the misfit of the aligner. With this, the patient did not have to frequently remove the elastics. Furthermore, the teeth had horizontal attachments that promoted aligner retention and were found to remain adjusted. Elastics were changed every day.

**Figure 6:**
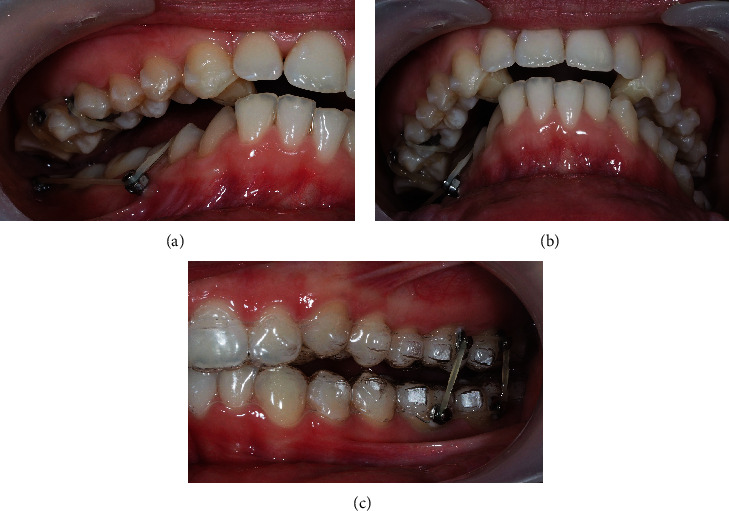
Removal of the occlusal ramps on teeth 36 and 37 and bite ramps placement in the upper canines. Vertical elastics on the left side to promote posterior intercuspation.

Primarily, bite ramps were placed to promote scissor bite disocclusion, first on the left side on the lower molars (Figure 5), but soon replaced for larger bite ramps on the palatal face of upper canines, just before the additional aligners (Figure 6). So, #35 of #45 total aligners were used on a first stage (Table 1).

**Figure 7:**
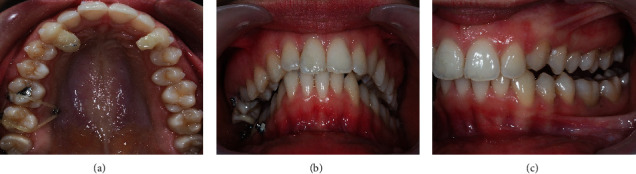
(a) Occlusal upper view; (b) overjet view; (c) left lateral photo to note the increased the left open bite due to the previous left posterior bite ramps, before the first additional aligners.

The lower retromolar MS has been lost, due to the upper right third molar interference, which was then removed, and other retromolar MS was placed in a more distal position to allow additional molar expansion and to continue to correct the deep bite on the scissor bite side. A vertical component elastic was used on the left side to promote posterior intercuspation and removed before first additional aligners (Figures 6 and 7).

**Figure 8:**
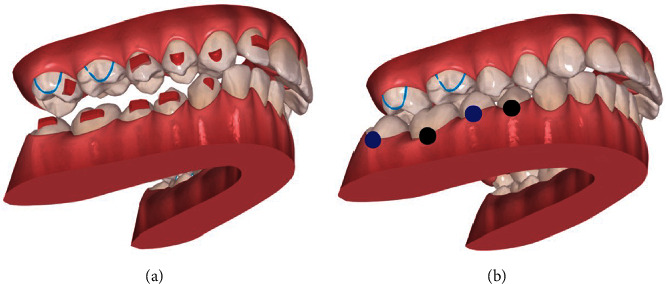
First additional aligners ClinCheck®: (a) beginning; (b) planned.

**Figure 9:**
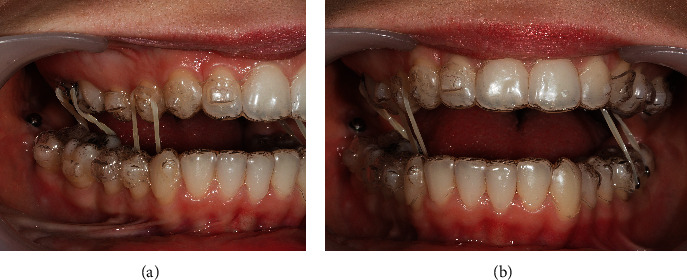
First additional aligners, with asymmetric crossed elastics.

In the first additional aligner stage (8 months after treatment began), crossed elastics were used on the right and left side, asymmetrically (Figures 8, 9 and Table 1).

**Figure 10:**
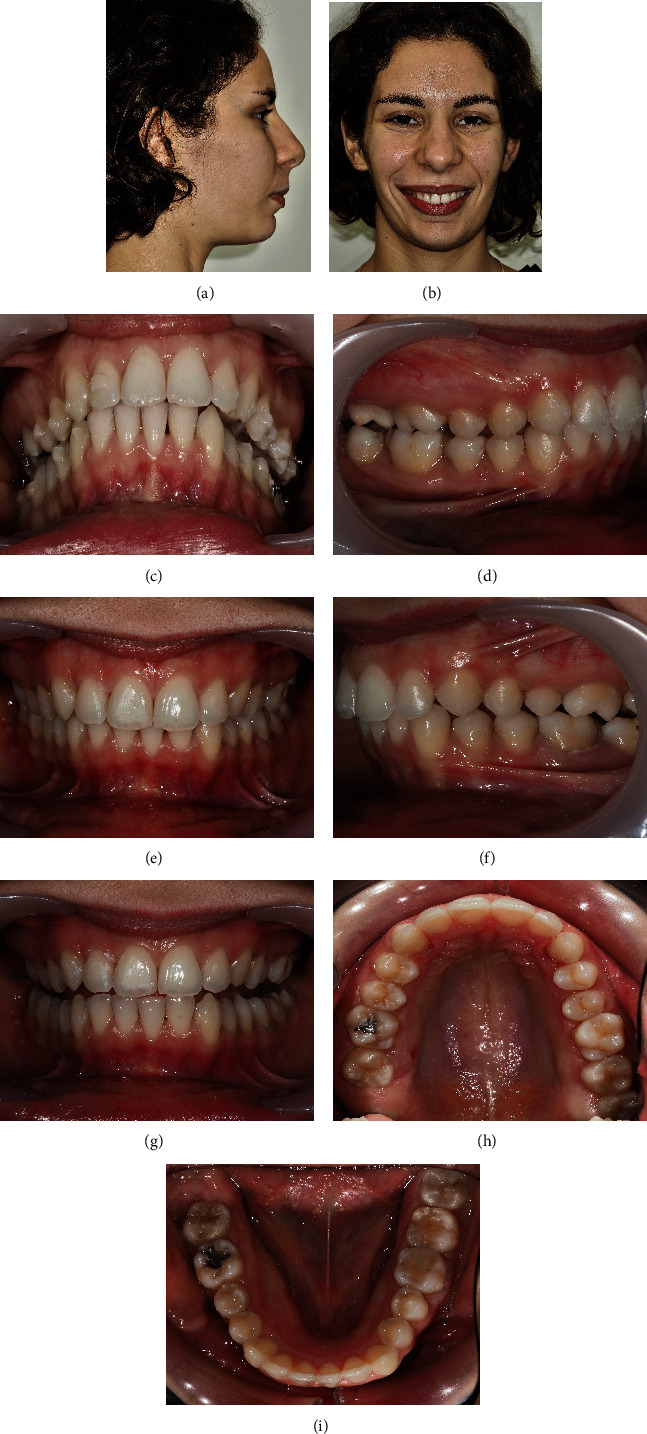
Photos of the end of 1st additional aligner stage, 1 year after the treatment beginning: (a) profile photo; b) smile; (c) overjet view; (d–f) intraoral photos in maximum intercuspation/centric relation; (g) protrusive guide; (h/i) occlusal photos.

**Figure 11:**
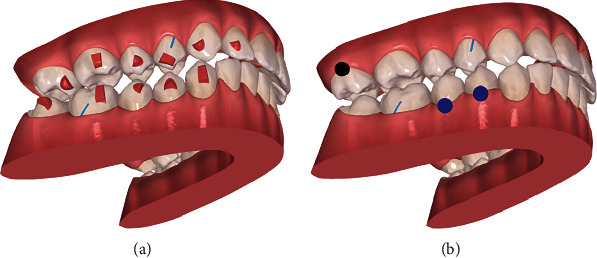
Second additional aligners ClinCheck®: (a) beginning; (b) planned.

**Figure 12:**
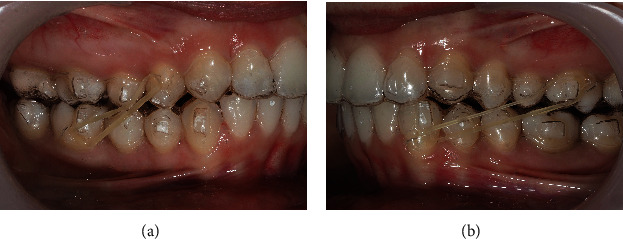
Removal of the MS: (a) class II elastics on the right side and (b) class III elastics on the left side to improve the asymmetry.

After the transversal and vertical improvement (Figure 10) and prior to a second additional aligner stage (Figure 11), all MS were removed and Class II elastics were placed on the right side, as well as a Class III elastics on the left side to stabilize the sagittal occlusion, even considering the lower dental asymmetry (Figure 12). In the second additional aligner stage, #48 of #52 aligners were used.

Three more additional aligners were needed to stabilize the occlusion between the upper and lower teeth, with the last aligner stage used only to stabilize before retention, used 12 h/day, at night, with all the 16 aligners used, changing them every month. All the information above is explained also on the checklist present in Table 1.

### 2.5. Treatment Results

After the two years and a half of active treatment, clear aligners with no attachments and movement velocity reduction were performed only using aligners to sleep, for one more year, to promote more stability to the case. Only then, upper and lower Vivera® retainers were placed.

**Figure 13:**
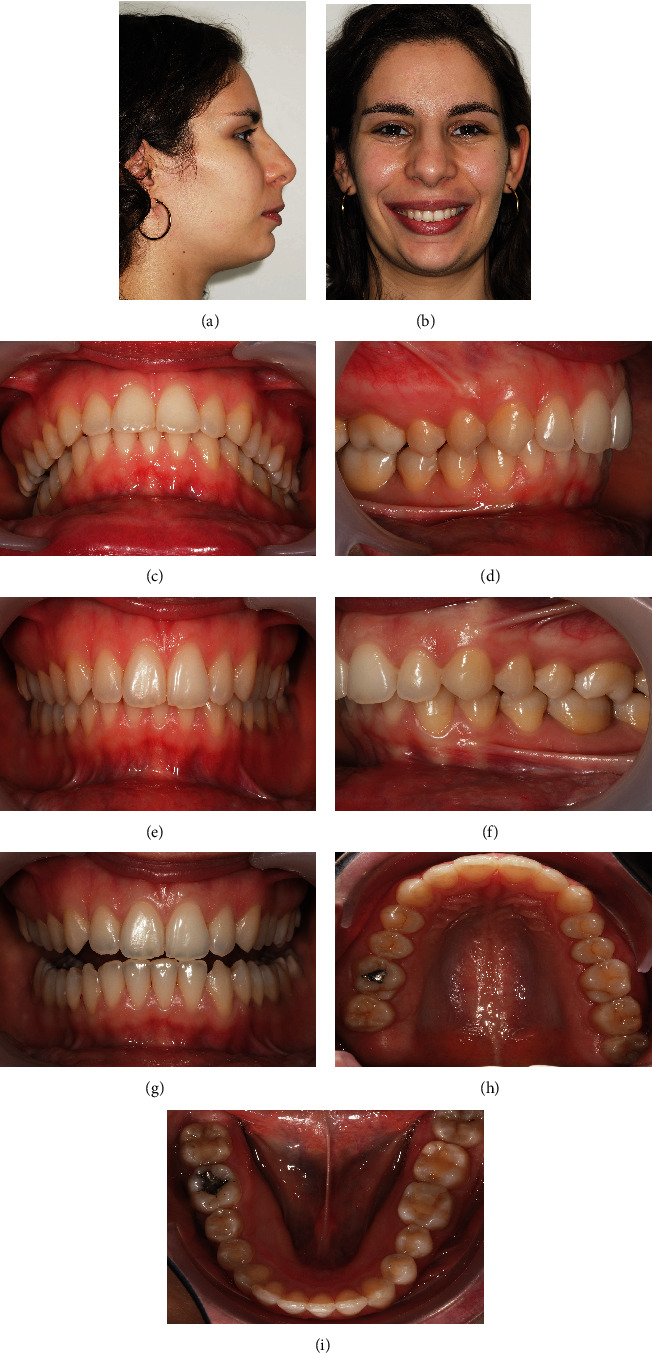
Final photos, 2 years after the treatment beginning: (a) profile photo; (b) smile; (c) overjet view; (d–f) intraoral photos in maximum intercuspation/centric relation; (g) protrusive guide; (h/i) occlusal photos.

At the treatment end, the scissor bite was successfully corrected. The stability achieved with the transverse and vertical orthodontically correction allowed the occlusal plane leveling. Despite the sagittal maintenance of the dental Class II on the right side, there was occlusal stability and improvement of the occlusion vertical dimension (Figure 13).

The sagittal positioning of the canines was maintained at the treatment end, thus maintaining the right Class II (Figure 13).

When looking at the mandibular base before and after orthodontic treatment, it can be seen the amount of alveolar remodeling that exists at the end, with a platform created lingually after correction of the high negative torque (Figure 13).

**Figure 14:**
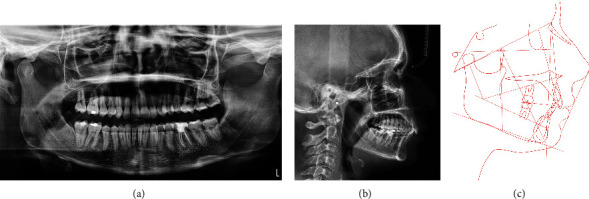
(a) Final panoramic X-ray, (b) teleradiograph, and (c) cephalometry.

Presence of the wisdom teeth only on the left side did not collide with our case stability (Figure 14(a)). On post-treatment cephalometric analysis, the following can be found: an hypodivergent biotype with similar value of FMA in the initial and final phase of treatment (FMA = 21.5° to FMA = 21.4°); pronounced skeletal Class II (convexity of the A point = 7.1 mm to convexity of the A point = 7.2 mm); class II at the ANB level (ANB = 7.2° to ANB = 7.7°); pronounced alveolar Class II (A-B distance = 9.8 mm to A-B distance = 10.6 mm), with normomaxilla where values do not change during treatment (SNA = 83.7° maintenance) and retromandible (SNB = 76.5° to SNB = 76.0°); and normal interincisal angle (129.7° to 129.2°), with accentuated retroclination of the upper incisors (UI/NA = 10.1° to UI/NA = 10.7°) and proclination of the lower incisors (IMPA = 105.4° to IMPA = 105.5°) (Figures 14(b) and 14(c)).

**Figure 15:**
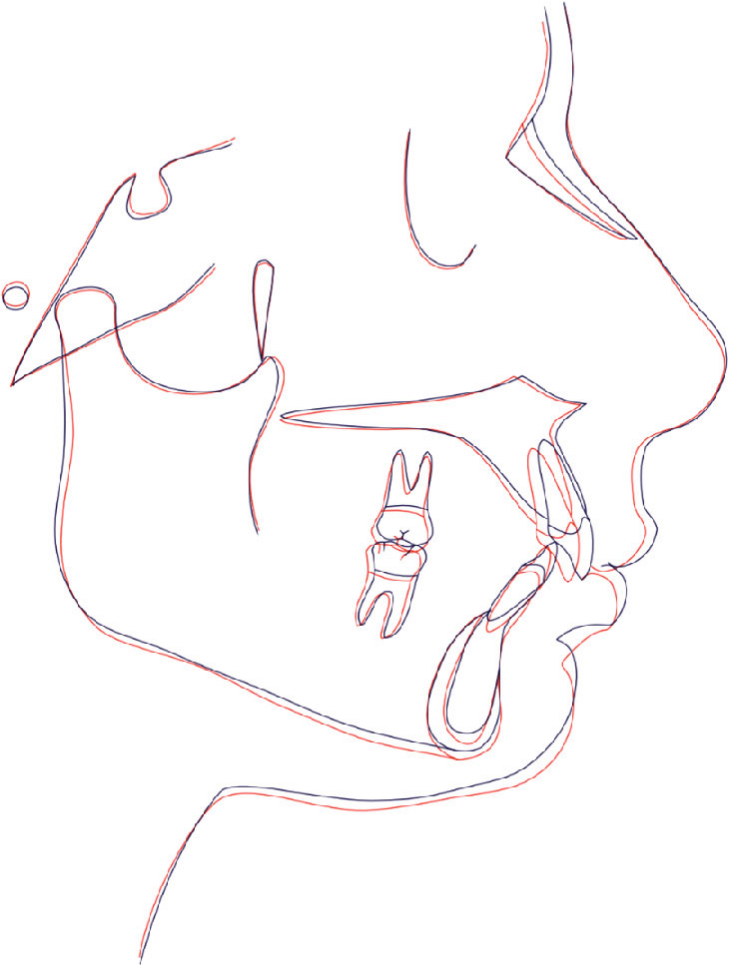
Superimposition of the initial tracing (black) and final tracing.

The cephalometric superimposition before and after treatment revealed a slight improvement in vertical and sagittal dimension (Figure 15).

The asymmetric lower sagittal dental position was stable, so the surgical treatment was not done, because to do so, it would be necessary to perform lower asymmetric extraction with much more tooth decompensation, with a longer treatment, and the problems associated to a surgical treatment.

The PAR index was approached in this case with two different authors (MLM and TP) and has been developed to provide a single summary score for the occlusal anomalies in a malocclusion. It estimates how far a case deviates from normal alignment and occlusion. The difference in scores between pre- and post-treatment reveals the degree of improvement. The PAR index shows uniformity and standardization in assessing the outcome of orthodontic treatment (Table 2) [11].

As a result of orthodontic treatment, the initial score has been reduced from 24 to 4, twenty points. The overall alignment has been improved in both arches, and the scissor bite in the right side and crossbite in the left side have been fully corrected.

**Figure 16:**
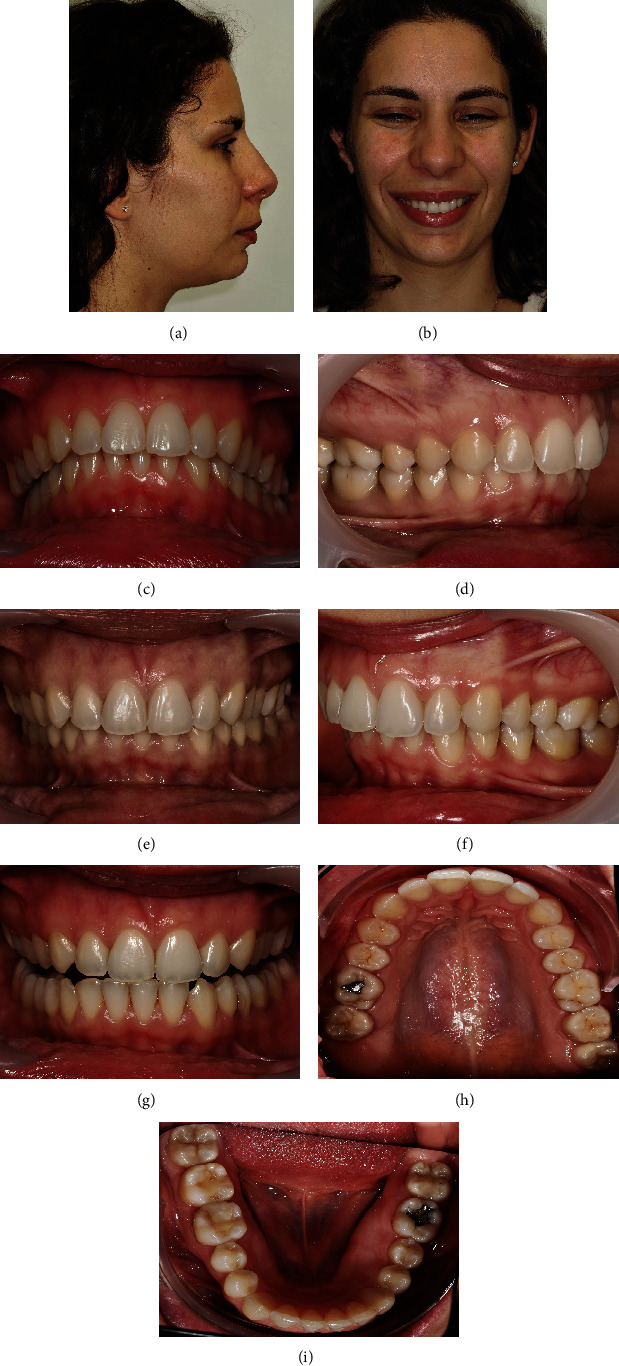
Follow-up photos, 2 years after the orthodontic treatment end: (a) profile photo; (b) smile; (c) overjet view; (d–f) intraoral photos in maximum intercuspation/centric relation; (g) protrusive guide; (h/i) occlusal photos.

In a 2-year follow-up, after orthodontic treatment, the stability of the case can be observed (Figure 16).

The clinical case was compared with a literature review portrayed in Table 3.

## 3. Discussion

To discuss this present clinical case, an integrative literature review was established on the existing data about the different treatment possibilities for scissor bite correction in adult patients. This review was performed in PubMed and BVS databases with the following conjugated keywords: “scissor bite OR brodie bite” AND malocclusion” AND “treatment OR correction OR therapeutics”, limited to the time from 2002 to March 2023 (Table 3).

Presently, scissor bite prevalence lies between 0.4 and 2.7% in adults [19]. As the growth slows down, the correction of this malloclusion becomes more difficult and with an increased necessity to perform orthodontic-surgical treatment [1, 7, 8, 20].

For patients that require surgery, its type depends on the etiology of the malocclusion, whether it is in the basal mandibular or maxillary arch [15]. However, surgical treatment is sometimes not easily accepted by patients as it is expensive and more invasive [21, 22].

Distraction osteogenesis has proven to be a very stable and predictable method in the scissor bite treatment, as demonstrated by Nascimento et al. [14] in their adult clinical case with bilateral scissor bite. It was also likely to improve functional, periodontal, and esthetic problems inherent to the scissor bite malocclusion. Furthermore, the distractor appliance used, proved to be minimally invasive, comfortable, economic, and easy to use [14, 23].

Distraction osteogenesis consists in the biological process of bone formation between preexisting bone segments that are progressively separated by controlled traction. The intermolar and intercanine distances usually remain stable after expansion, and healing/stability usually occurs within 3 to 4 months, so that the distraction device can be removed and orthodontic treatment subsequently initiated [5, 14].

Sakamoto et al. [5] demonstrated the effectiveness of lateral mandibular expansion treatment after corticotomy in a young adult patient with a skeletal scissor bite. According to these authors, expansion by corticotomy reduces the gingival recession risk and hyperesthesia and also guarantees post-treatment stability. It also has minor effect on the temporomandibular joint and a lower chance of tooth damage [5].

Subapical mandibular surgeries have been used to correct vertical malocclusion and problems associated with mandibular deformity [13]. Posterior subapical mandibular surgery compared to anterior and total mandibular surgery has been used less frequently, mainly because it involves a higher risk of injury of the inferior alveolar neurovascular bundles. However, it can be done successfully with proper incisional design and a careful surgical technique [5, 13]. Suda et al. [13] verified the effectiveness of posterior subapical mandibular surgery in correcting successfully the scissor bite and collapsed mandibular arch in an adult patient. The treatment also included Le Fort I osteotomy and sagittal split ramus osteotomy (SSRO). This patient had a satisfactory outcome with no sensory or motor paralysis after surgery, demonstrating the efficiency of this surgical approach without complications or problems when the surgery is well planned [13].

The 3-piece Le Fort I osteotomy is a suitable treatment for reducing the bilateral or unilateral maxillary width while maintaining the intercanine width. In addition, it allows a good access to the sagittal suture, being a procedure that requires only moderate patient cooperation, and reduces the total treatment time [8, 15].

Kim et al. [15] and Morelon et al. [8] described Le Fort I osteotomy as an effective, acceptable, and rapid solution for the treatment of scissor bite. Kim et al. [15] also reported the combination of Le Fort I osteotomy with sagittal split ramus osteotomy (SSRO) as an effective treatment to improve facial asymmetry as well as transverse discrepancy.

The results obtained in the present clinical case with a high complexity, since it was a total unilateral scissor bite with many dental compensations in the 3 planes of space and consequent inherent skeletal repercussions, using an initial approach of Invisalign® aligners, bite ramps, and MS, were compared with those from publications using different orthodontic approaches in adult patients.

Less invasive procedures, such as MS, are particularly suitable for the treatment of severe scissor bite in adult patients without side effects [6]. Orthodontic MS have become very popular for absolute anchorage during various types of tooth movement since they have more advantages than conventional dental implants, such as favorable biomechanical properties and the possibility of placement in various anatomical sites such as the alveolar bone between the roots of the teeth and in the palate due to sufficient cortical bone thickness [4, 7].

Clear aligners and braces are reported to be both an effective option in treating mild to severe malocclusion. Aligners had advantage in segmented movement of teeth, something pertinent in this case, but may not be as effective as braces in producing adequate occlusal contacts [24]. The posterior inocclusion promoted by the aligner plastic between the arches was not enough to promote the transversal movement to correct the present scissor bite, due to the deep bite severity. So, the bite ramps were an essential auxiliary to allow enough right posterior inocclusion to overcome the dental compensations. Also, the MS were an important auxiliary due to the movement's unpredictability [21].

Note that, despite aligners being a more comfortable/esthetic approach, the auxiliaries (MS, bite ramps, and elastics) used essentially during the first year of treatment, were not so pleasant. However, lower brackets if used, the same auxiliaries would be necessary and used in this treatment approach. Additionally, lower brackets would fall off very often because of its interferences with occlusal surfaces of upper teeth, due to the severity of the deep bite on the scissor bite condition [25].

Jung [7] and Kim et al. [15] also proved in their clinical cases in adult patients with skeletal and dental scissor bite, respectively, the effectiveness of MS with no reported side effects or relapse. Furthermore, Nakamura et al. [17] also found that the combination of MS and fixed bite blocks was effective and efficient in facilitating the correction of bilateral skeletal scissor bite.

There are two types of MS: intra- and extra-alveolar. The intra-alveolar (interradicular ones) are commonly used as skeletal anchorage because they are relatively easy to place and provide direct anchorage to intrude teeth. However, Baik et al. [6] found that interradicular MS are more effective in the maxilla than in the mandible, where they have high failure rates.

On the other hand, extra-alveolar MS have a prominent head to retain the elastic chains and allow a position up to 10 mm from the buccal face of inclined molars, with positioning of the head more buccally, and deeply, if a more intrusive force component is required. Also, extra-alveolar MS associated with glass ionomer bite tubes have been reported as a treatment option by Lee et al. [16] who found it to be a minimally invasive combination, allowing the improvement of the skeletal malocclusion in a short period of time without patient cooperation.

The combined use of palatal MS and lingual multibracket appliances also increases the efficiency of molar scissor bite correction, as demonstrated by Tamamura et al. [4] providing esthetics, as required by the patient.

However, future studies should be conducted on other important aspects, such as miniscrew diameter [26], geometric design [27], and damages to surrounding tissues [28], to allow safer treatment in combination with both multibracket and aligner appliances.

Currently, there is a growing demand for esthetic treatments among adolescents and adults, as is the case described in the literature, of a 21-year-old patient who wanted to correct her dental scissor bite, but without using fixed orthodontics appliances [18]. For the correction of the scissor bite only on the right side between the first premolars, associated with a deep bite, Habash [18] was the only one who used the Invisalign® system as a treatment method.

The aligners, made of transparent thermoplastic polymer, allow a maximum of 0.25 mm, or 2 degrees per rotation, or 1 degree for lingual root torque in each aligner [29]. They must be worn at least 22 hours a day and have to be replaced every 10 or 7 days [30, 31]. The possibility of removing these aligners also allows the patient to have better daily control of oral hygiene.

In our case, even though the scissor bite was complex and involved a large number of teeth, unlike Habash [18] who only had 2 teeth involved, it can be shown that the use of clear aligners is an effective way to solve orthodontic problems such as scissor bite and crowding in a time frame comparable to conventional fixed orthodontics. However, in these complex cases, auxiliaries such as bite ramps and intra- and inter- arch elastics associated with MS, were essential.

Chugh et al. [12] used cross elastics in combination with a maxillary bite plate, supported on the anterior teeth, in the correction of a bilateral dental scissor bite, without clear aligners. These authors concluded that the malocclusion was successfully corrected and that, if the patient is reasonably motivated, orthodontic therapy in adults can provide complete rehabilitation in both function and appearance with a satisfactory prognosis.

Concerning about the PAR index that was taken in this case, and as described in the literature, the amount of reduction in PAR score reflects the degree of improvement and the treatment success [11].

The present severe clinical case was treated only orthodontically since after the SB correction the occlusion in the 3 planes of the space was stable, even with the presence of the asymmetric lower dental sagittal position.

The severe limitations to decompensating tooth positions for a surgical treatment, with the necessity to perform lower asymmetric extraction and a must longer orthodontic treatment, were the major reasons to avoid the surgery after a first stage of descompensation [19, 32]. Also, the difficulty degree associated with an hypodivergent biotype, with a severe deep bite, was very limitative to solve this scissor bite. It should be noted that the patient had a crossbite evolving a total unilateral arch, being that the reason for using auxiliaries to improve the unpredictable dental compensation movements that the patient presented at the beginning.

## 4. Conclusions

In adult patients with severe facial asymmetry or basal arch width discrepancy, given the lack of growth, a surgical approach may be the best option. Within the surgical approaches, we have distraction osteogenesis, corticotomy, subapical mandibular surgery, and Le Fort I osteotomy.

Less invasive procedures such as MS, associated or not with bite ramps, are particularly suitable for the treatment of severe scissor bite without side effects. The association of clear aligners, MS, bite ramps, and intra- and inter-arch elastics was fundamental for the success achieved in the correction of this severe scissor bite malocclusion case.

## Figures and Tables

**Table 1:** Procedures followed in the present case.

Stage	Procedure	Aligners used
Initial	(1) Upper arch—aligners and one palatal MS (inter-radicular between teeth 1.6 and 1.7), with 8 mm (Kubident®, Spain) to connect an elastic (1/4^″^ 6 oz) to buccal buttons on teeth 1.6 and 1.7 Lower arch—two MS on the 4th quadrant: one on the retromolar trigone (14 mm, Kubident®); one inter-radicular between the right premolars buccally (10 mm, Kubident®), associated with elastics (1/4^″^ 3.5 oz) to lingual buttons on teeth 47, 46, 45, and 44 (i) Posterior bite ramps on teeth 3.6 and 3.7; aligners cut on occlusal surface to readapt to these bite ramps (2) 6 months after (i) Removal of the bite ramps on teeth 3.6 and 3.7 (ii) New large bite ramps on the upper canines; aligners cut on occlusal surface to readapt to these bite ramps (iii) Maintenance of the same elastics method, with the addition of elastic (3/16^″^ 4.5 oz) between buttons on buccal surfaces of teeth 2.6/2.7 and 3.6/3.7 (3) 2 months after the canine bite ramps placement (i) New aligner request	35 of 45

1st additional aligners	(1) Asymmetric crossed elastics: on the right side, elastics (1/4^″^ 3.5 oz) between teeth (2) Class II elastics were placed on the right side, as well as a class III component on the left side to stabilize the sagittal occlusion, even considering the lower dental asymmetry (3) All the MS were removed at the end of this stage	32 of 42

2nd additional aligners	(4) Maintenance of the canine bite ramps and asymmetric crossed elastics: on the right side, elastics (1/4^″^ 3.5 oz) between teeth (5) Sequential distalization on the 1st quadrant and mesialization of the 4th quadrant, also reinforced by the elastic, in order to stabilize the asymmetric occlusion (6) Extrusion of the posterior sectors, to promote intercuspation/occlusal contacts and anterior intrusion	48 of 52

3rd additional aligners	(7) Extrusion of the posterior sector to promote intercuspation/occlusal contacts and anterior intrusion, mainly on the upper arch	13 of 13

4th additional aligners	(8) Reinforcement of the posterior sector extrusion to promote intercuspation/occlusal contacts and anterior intrusion, mainly on the upper arch	27 of 27

5th additional aligners	(9) Reinforcement of the posterior sector extrusion to promote intercuspation/occlusal contacts and anterior intrusion, mainly on the upper arch (10) Removal of all the attachments	13 of 13

6th additional aligners	(11) Aligners used 12 h/day at night and changed once a month, to stabilize prior to the Vivera® retention	16 of 16

**Table 2:** PAR index.

PAR components	Before treatment	Total	After treatment	Total
Upper right segment	Discrepancy between 0 mm and 1 mm	0	Discrepancy between 0 mm and 1 mm	0
Upper anterior segment	Discrepancy between 4.1 mm and 8 mm	3	Discrepancy between 0 mm and 1 mm	0
Upper left segment	Discrepancy between 1.1 mm and 2 mm	1	Discrepancy between 0 mm and 1 mm	0
Lower right segment	Discrepancy between 1.1 mm and 2 mm	1	Discrepancy between 0 mm and 1 mm	0
Lower anterior segment	Discrepancy between 4.1 mm and 8 mm	3	Discrepancy between 0 mm and 1 mm	0
Lower left segment	Discrepancy between 1.1 mm and 2 mm	1	Discrepancy between 0 mm and 1 mm	0
Right buccal occlusion	Half a unit discrepancy (cusp to cusp) 2 More than one tooth in scissor bite 4	6	Good interdigitation classes I, II, and III 0 No discrepancy in intercuspation 0 No crossbite 0	0
Overjet	Overjet between (5.1–7 mm) 5.3 mm	2	Overjet between (5.1–7 mm) 5,4 mm	2
Overbite	Greater than two-thirds coverage of the lower incisors	2	Greater than one-thirds but less than two-thirds coverage of the lower incisors	1
Centerline	One-quarter to one-half lower incisor width	1	One-quarter to one-half lower incisor width	1
Left buccal occlusion	Less than half unit discrepancy 1 Lateral open bite on at least two teeth greater than 2 mm 1 Single tooth in crossbite 2	4	Good interdigitation classes I, II, and III 0 No discrepancy in intercuspation 0 No crossbite 0	0
Total		24		4

**Table 3:** Relevant results of the selected articles.

Author and year of publication	Title	Sample	Etiology	Treatment	Conclusion
Presented case	“Scissor bite: systematic review and clinical case treated with aligners and mini-implants”	♀ 24 years old SB—total right side (14, 15, 16, 17/44, 45, 46, 47) Other associated problems: convex profile and facial asymmetry; deep bite with pronounced cant in the lower occlusal plane on the SB side	SB—dental+skeletal (mandible); severe compression of the mandibular arch; right molar and canine class II; left molar and canine class III; skeletal class II, hypodivergent	*Maxilla*: Invisalign aligner series (22 h/day)+buccal button of 17 and palatal MS between 16 and 17 *Mandible*: Invisalign aligner series (22 h/day)+lingual button 44, 45, 46, and 47 and MS in retromolar trigone and between 44 and 45 *Total treatment time*: 1 year 8 months *Retainers*: Vivera® *Control*: 2 years, no relapse	Integration of aligners and MS can be effective to orthodontically treat severe SB with occlusal plane control in adult patients Despite the maintenance of the class II relationship on the right side, there is a stable occlusion and improvement of the vertical dimension

Tamamura et al. [4]	“Use of palatal miniscrew anchorage and lingual multi-bracket appliances to enhance efficiency of molar scissor-bite correction”	♀ 17 years and 4 months SB—right side (17/47) Other associated problems: straight profile without facial asymmetry; crowding of the upper and lower front teeth	SB—dental; bilateral molar class I; bilateral canine class I; skeletal class I	*Maxilla*: 2 MS in the palatal region of the 17 (1 buccal, 1 palatal)+elastic chain by occlusal 17+lingual multibracket appliance *Mandible*: buccal brackets appliance *Total treatment time*: 26 months *SB correction*: 3 months *Retainer*: wraparound for maxilla and lingual adhesive retainer for mandible	Combination of palatal MS and lingual multibracket appliances increases the efficiency of SB correction in molars

Chugh et al. [12]	“Brodie bite with an extracted mandibular first molar in a young adult: a case report”	♀ 17 years old SB—bilateral posterior Other associated problems: absence of 46; convex profile without facial asymmetry	SB—dental; left molar class I; right molar class II; bilateral canine class I; skeletal class I	*Maxilla*: maxillary bite plate on anterior teeth (3 months) *Mandible*: stainless steel jockey arch with crossed elastics *Total treatment time*: 18 months *SB correction*: 5 and a half months *Retainer*: maxilla: Hawley with labial arch; mandible: lingual retainers bonded canine-to-canine	Conventional orthodontic treatment is a minimally invasive option that can, depending on the patient, provide a complete rehabilitation with a satisfactory prognosis

Jung [7]	“Treatment of severe scissor bite in a middle-aged adult patient with orthodontic mini-implants”	♂ 49 years old SB—left side (24, 25, 26, 27/34, 35, 36, 37, 38) Other associated problems: straight profile, slight facial asymmetry, and deviation of the mandible on the right side	SB—skeletal (mandible); right and left canine class II; indeterminate bilateral molar class; skeletal class II	*Maxilla*: ceramic brackets buccally on teeth 24, 25, 26, and 27. MS on the palatal and buccal alveolar bone. Metallic buttons on the palatal face of the 24, 25, 26, and 27 with elastic chain *Mandible*: ceramic brackets lingually on teeth 34, 35, 36, and 37 1 MS between teeth 35 and 36 *Total treatment time*: 2 years *SB correction*: 18 months *Retainer*: mandible—Hawley retainer; maxilla—circumferential retainer *Control*: 14 months, lower molars with slight lingual tipping and distal face of 27 with greater pocket depth (lost retention)	The final records showed that orthodontic MS were very effective in correcting SB

Suda et al. [13]	“Orthognathic treatment for a patient with facial asymmetry associated with unilateral scissors-bite and a collapsed mandible arch”	♀ 21 years old SB—right side (14, 15, 16, 17/44, 45, 46, 47) Other associated problems: facial asymmetry and unilateral collapsed mandibular arch (right)	SB—skeletal (mandible); bilateral class I; skeletal class II	*Maxilla*: bite plate *Mandible*: posterior subapical mandibular surgery and lingual arch *Total treatment time*: 4 years and 8 months *SB correction*: 7 months *Retainer*: Hawley circumferential retainers in the maxilla and mandible *Control*: 1 year, no relapse	Efficacy of posterior subapical mandibular surgery in the successful correction of scissor bite and collapsed mandibular arch

Nascimento [14]	“Treatment of bilateral Brodie bite in a periodontally compromised patient using distraction osteogenesis”	♂ 37 years old SB—bilateral posterior Other associated problems: concave profile without asymmetry	SB—skeletal (mandible); class II division 2; skeletal class II	*Maxilla*: fixed appliance *Mandible*: distraction osteogenesis in the mandibular symphysis region+mandibular distraction device with palatal screw (13 days of activation and 90 days of stabilization)+mandibular advancement surgery *Retainer*: circumferential in the maxilla and lingual canine-to-canine retainer in the mandible	Distraction osteogenesis of the mandibular symphysis has proven to be very important in the treatment of bilateral SB, making it possible to correct functional, periodontal, and esthetic problems

Kim et al. [15]	“Surgery versus nonsurgery option for scissors bite treatment”	Case 1: ♂ 36 Y SB—left side (24, 25, 26, 27, 28/34, 35, 36, 37, 38)	Case 1: SB—dental; mild skeletal class III	Case 1: *Maxilla*: fixed appliance with 2 MS; metallic buttons on palatal surface of posterior teeth for elastic chain *Mandible*: fixed appliance with 1 MS in the buccal alveolar bone between teeth 35 and 36	Case 1: MS can be used to successfully correct SB.
		Case 2: ♂ 28 Y SB—posterior bilateral Other associated problems: facial asymmetry	Case 2: SB—skeletal severe (maxilla); bilateral class III; skeletal class I	Case 2: *Maxilla*: Le Fort I maxillary osteotomy *Mandible*: SSRO and mentoplasty *SB correction*: 24 months	Case 2: the combination of Le Fort I segmental osteotomy and SSRO was an effective treatment to improve facial asymmetry as well as transversal discrepancy

Sakamoto et al. [5]	“Bilateral scissor bite treated by rapid mandibular expansion following corticotomy”	♂ 17 Y SB—bilateral posterior Other associated problems: convex profile and prognathism of the maxilla	SB—skeletal (mandible); right molar class I; left class I; skeletal class II	*Maxilla*: quad-helix (expansion of 16 and 26) *Mandible*: corticotomy and rapid lateral mandibular expansion appliance (36, 33, 43, 46) *Total treatment time*: 4 years and 7 months *SB correction*: 7 months *Retainers*: circumferential in the maxilla and mandible *Control*: 2 years and 7 months, no relapses	Lateral mandibular expansion after corticotomy is effective in young adult patients with a narrow mandibular arch

Morelon et al. [8]	“Traitement d'un syndrome de Brodie unilatéral par contraction chirurgicale des maxillaires”	♂ 22 Y SB—left side (24, 25, 26, 27, 28/34, 35, 36, 37, 38) Other associated problems: maxillary prognathism	SB—skeletal (maxilla); right molar class I; right canine class I; skeletal class II	*Maxilla*: segmented Le Fort I osteotomy fixed appliance 15 days after osteotomy *SB correction*: 6 months *Retainer*: 4 osteosynthesis plates in “L” and “J” shape	Le Fort I osteotomy is an acceptable and quick solution for the treatment of SB

Lee et al. [16]	“Severe unilateral scissors-bite with a constricted mandibular arch: bite turbos and extra-alveolar bone screws in the infrazygomatic crests and mandibular buccal shelf”	♀ 33 Y SB—right side (14, 15, 16, 17/44, 45, 46, 47) Other associated problems: convex profile; mandibular arch constriction and facial asymmetry	SB—skeletal (mandible); left molar class I; left canine class I; skeletal class II	Maxilla: fixed appliance with 2 occlusal bite tubes cemented on teeth 26 and 27 and extra-alveolar MS on the infrazygomatic ridge (retract the maxillary arch) *Mandible*: fixed appliance with lingual brackets on teeth 46 and 47+extra-alveolar buccal MS between 46 and 47 *Total treatment time*: 27 months *SB correction*: 4 months *Retainer*: invisible overlay retainers for both arches *Control*: 38 months, no relapse	Extra-alveolar MS are a minimally invasive method for SB correction with maxillary protrusion

Baik et al. [6]	“Correcting severe scissor bite in an adult”	♀ 28Y SB—right side (14, 15, 16, 17/44, 45, 46, 47) Other associated problems: convex profile and facial asymmetry	SB—dental+skeletal (mandible); left molar and canine class I; undetermined right molar and canine class; skeletal class II, hyperdivergent	*Maxilla*: fixed appliance with 2 interradicular MS, 1 buccal and, 1 palatal between the 16 and 17+removable bite plate *Mandible*: modified lingual arch *Total treatment time*: 3 years *SB correction*: 7 months *Retainer*: wraparound removable appliance *Control*: 6 years, no relapse	Integration of MS and lingual arch may be effective for treating severe SB in adult patients

Nakamura et al. [17]	“Nonsurgical orthodontic treatment of a hypodivergent adult patient with bilateral posterior scissors bite and excessive overjet”	♀ 26 Y SB—bilateral posterior Other associated problems: convex profile; excessive overjet	SB—skeletal (narrow mandible, wide maxilla); class II division 1; skeletal class I, hypodivergent	*Maxilla*: lingual arch with an anterior bite block, 2 MS (1 between 24 and 25 and 1 between 26 and 27, both in palatal)+posterior edgewise appliance with elastics *Mandible*: 2 MS (1 between 34 and 35 and 1 between 36 and 37, both buccally)+edgewise appliance with elastics+lingual arch *Total treatment time*: 56 months *SB correction*: 12 months *Retainer*: removable and fixed in both arches *Control*: 13 months, no relapses	Fixed MS and bite blocks were effective and efficient in facilitating the correction of bilateral SB

Habash [18]	“Scissor bite and crowding correction with clear aligners: case report”	♀ 21 Y SB—right side (14/44) Other associated problems: deep bite	SB—dental; bilateral molar and canine class I; skeletal class I	*Maxilla*: Invisalign aligner series (22 h/day) *Mandible*: Invisalign aligner series (22 h/day) *Total treatment time*: 14 months *Retainer*: fixed and removable (not specified) *Control*: 1 year, no relapse	The use of aligners is an effective way of solving orthodontic problems such as SB and crowding in a time frame comparable to conventional fixed orthodontics. In addition, this system is associated with excellent oral hygiene and esthetics

SB: scissor bite; MS: miniscrew; TPA: transpalatal arch; DB: distobuccal.

## References

[1] Pinho T. (2011). Early treatment of scissor bite. *Journal of Clinical Orthodontics*.

[2] Nojima K., Takaku S., Murase C., Nishii Y., Sueishi K. (2011). A case report of bilateral Brodie bite in early mixed dentition using bonded constriction quad-helix appliance. *The Bulletin of Tokyo Dental College*.

[3] Deffrennes G., Deffrennes D. (2017). Management of Brodie bite: note on surgical treatment. *International Orthodontics*.

[4] Tamamura N., Kuroda S., Sugawara Y., Takano-Yamamoto T., Yamashiro T. (2009). Use of palatal miniscrew anchorage and lingual multi-bracket appliances to enhance efficiency of molar scissors-bite correction. *The Angle Orthodontist*.

[5] Sakamoto T., Hayakawa K., Ishii T., Nojima K., Sueishi K. (2016). Bilateral scissor bite treated by rapid mandibular expansion following corticotomy. *The Bulletin of Tokyo Dental College*.

[6] Baik U. B., Kim Y., Sugawara J., Hong C., Park J. H. (2019). Correcting severe scissor bite in an adult. *American Journal of Orthodontics and Dentofacial Orthopedics*.

[7] Jung M. H. (2011). Treatment of severe scissor bite in a middle-aged adult patient with orthodontic mini-implants. *American Journal of Orthodontics and Dentofacial Orthopedics*.

[8] Morelon J. B., Meyer C., Parmentier J., Prost G., Weber E., Louvrier A. (2017). Treatment of a unilateral Brodie's syndrome by surgical contraction of the maxillae. *Journal of Stomatology, Oral and Maxillofacial Surgery*.

[9] Pinho T., Gonçalves S., Rocha D., Martins M. L. (2023). Scissor bite in growing patients: case report treated with clear aligners. *Children*.

[10] Lee K. M., Lim S. H., Lee G. H., Park J. H. (2020). *Scissor Bite Correction with TADs*.

[11] Richmond S., Shaw W. C., O'Brien K. D. (1992). The development of the PAR index (peer assessment rating): reliability and validity. *European Journal of Orthodontics*.

[12] Chugh V. K., Sharma V. P., Tandon P., Singh G. P. (2010). Brodie bite with an extracted mandibular first molar in a young adult: a case report. *American Journal of Orthodontics*.

[13] Suda N., Tominaga N., Niinaka Y., Amagasa T., Moriyama K. (2012). Orthognathic treatment for a patient with facial asymmetry associated with unilateral scissors-bite and a collapsed mandibular arch. *American Journal of Orthodontics*.

[14] Nascimento L. E. (2013). Treatment of bilateral Brodie bite in a periodontally compromised patient using distraction osteogenesis. *Journal of the World Federation of Orthodontists*.

[15] Kim K. A., Yu J. J., Chen Y., Kim S. J., Kim S. H., Nelson G. (2015). Surgery versus nonsurgery option for scissors bite treatment. *The Journal of Craniofacial Surgery*.

[16] Lee S. A., Chang C. C. H., Roberts W. E. (2018). Severe unilateral scissors-bite with a constricted mandibular arch: bite turbos and extra-alveolar bone screws in the infrazygomatic crests and mandibular buccal shelf. *American Journal of Orthodontics*.

[17] Nakamura M., Kawanabe N., Adachi R., Yamashiro T., Kamioka H. (2019). Nonsurgical orthodontic treatment of a hypodivergent adult patient with bilateral posterior scissors bite and excessive overjet. *The Angle Orthodontist*.

[18] Habash A. A. (2020). Scissor bite and crowding correction with clear aligners: case report. *Webmed Central Orthodontics*.

[19] Tomonari H., Kubota T., Yagi T. (2014). Posterior scissors-bite: masticatory jaw movement and muscle activity. *Journal of Oral Rehabilitation*.

[20] Pinho T., Figueiredo A. (2011). Orthodontic-orthognathic surgical treatment in a patient with class II subdivision malocclusion: occlusal plane alteration. *American Journal of Orthodontics*.

[21] Pinho T., Santos M. (2021). Skeletal open bite treated with clear aligners and miniscrews. *American Journal of Orthodontics*.

[22] Pinho T., Rocha D. (2021). Asymmetrical skeletal class III camouflage treatment with clear aligners and miniscrew anchorage. *Journal of Clinical Orthodontics*.

[23] King J. W., Wallace J. C. (2004). Unilateral Brodie bite treated with distraction osteogenesis. *American Journal of Orthodontics*.

[24] Marcelino V., Baptista S., Marcelino S. (2023). Occlusal changes with clear aligners and the case complexity influence: a longitudinal cohort clinical study. *Journal of Clinical Medicine*.

[25] Ke Y., Zhu Y., Zhu M. (2019). A comparison of treatment effectiveness between clear aligner and fixed appliance therapies. *BMC Oral Health*.

[26] Sfondrini M. F., Gandini P., Alcozer R., Vallittu P. K., Scribante A. (2018). Failure load and stress analysis of orthodontic miniscrews with different transmucosal collar diameter. *Journal of the Mechanical Behavior of Biomedical Materials*.

[27] Radwan E. S., Montasser M. A., Maher A. (2018). Influence of geometric design characteristics on primary stability of orthodontic miniscrews. *Journal of Orofacial Orthopedics*.

[28] Montasser M. A., Scribante A. (2022). Root injury during interradicular insertion is the most common complication associated with orthodontic miniscrews. *The Journal of Evidence-Based Dental Practice*.

[29] D'Antò V., Bucci R., De Simone V., Huanca Ghislanzoni L., Michelotti A., Rongo R. (2022). Evaluation of tooth movement accuracy with aligners: a prospective study. *Materials*.

[30] Al-Nadawi M., Kravitz N. D., Hansa I., Makki L., Ferguson D. J., Vaid N. R. (2021). Effect of clear aligner wear protocol on the efficacy of tooth movement. *The Angle Orthodontist*.

[31] Fry R. (2017). Weekly aligner changes to improve Invisalign treatment efficiency. *Journal of Clinical Orthodontics*.

[32] Agrawal A. (2020). Brodie bite: a clinical challenge. *International Journal of Clinical Pediatric Dentistry*.

